# Substance Use Disorder-Related Deaths and Maternal Mortality in New Mexico, 2015–2019

**DOI:** 10.1007/s10995-023-03691-8

**Published:** 2023-06-12

**Authors:** Jessica R. Fuchs, Melissa A. Schiff, Eirian Coronado

**Affiliations:** 1 New Mexico Department of Health, 2040 S. Pacheco St, Santa Fe, NM 87505 USA; 2 Department of Internal Medicine, University of New Mexico School of Medicine, 1 University of New Mexico, Albuquerque, NM 8713 USA

**Keywords:** Maternal mortality, Substance use, Mental health, Pregnancy, Postpartum

## Abstract

**Background:**

In recent decades, maternal mortality has increased across the U.S. Experiences of pregnant and postpartum people with Substance Use Disorder (SUD)-related deaths have not been previously evaluated in New Mexico. The aim of this study was to analyze risk factors related to substance use and explore substance use patterns among pregnancy-associated deaths in New Mexico from 2015 to 2019.

**Methods:**

We conducted an analysis of pregnancy-associated deaths to assess the association between demographics, pregnancy factors, circumstances surrounding death, treatment of mental health conditions, and experiences with social stressors among Substance Use Disorder (SUD)-related and non-SUD-related deaths. We performed univariate analyses of risk factors using chi-square tests to assess the differences between SUD-related and non-SUD-related deaths. We also examined substance use at time of death.

**Results:**

People with SUD-related deaths were more likely to die 43–365 days postpartum (81% vs. 45%, p-value = 0.002), have had a primary cause of death of mental health conditions (47% vs 10%, p < 0.001), have died of an overdose (41% vs. 8%, p-value = 0.002), have had experienced any social stressors (86% vs 30%, p < 0.001) compared to people with non-SUD-related deaths, and received treatment for SUD at any point before, during, or after pregnancy (49% vs. 2%, p < 0.001). The substances most used at time of death were amphetamines (70%), and most cases engaged in polysubstance use (63%).

**Conclusions for Practice:**

Providers, health departments, and community organizations must prioritize supporting people using substances during and after pregnancy to prevent death and improve quality of life for pregnant and postpartum people.

## Significance

*What is Already Known on this Subject?* Maternal mortality rates have been increasing across the U.S. Substance use and overdoses are leading causes of death among pregnant and postpartum people. Return to use in the postpartum period following abstinence during pregnancy is common. New Mexico has some of the highest rates of death attributed to alcohol use, and experiences high rates of substance use.

*What does this study add?* This study provides background on risk factors for Substance Use Disorder (SUD)-related death among pregnant and postpartum people in New Mexico. It explores experiences with mental health treatments and social stressors, and substance use patterns among pregnant and postpartum people with SUD-related deaths.

## Background

In recent decades, maternal mortality has increased significantly across the U.S. The Centers for Disease Control and Prevention (CDC) has launched efforts to combat these increases through jurisdiction-level Maternal Mortality Review Committees (MMRCs) to comprehensively review pregnancy-associated deaths and make recommendations to prevent future deaths (CDC, 2022a). MMRCs have strengthened surveillance efforts to track leading causes of pregnancy-associated deaths. Substance use has been identified as a leading cause of pregnancy-associated deaths (Smoots et al., 2021).

A review of 22 states from 2007 to 2016 found that pregnancy-associated mortality involving opioids more than doubled, from 1.3 deaths to 4.2 deaths per 100,00 live births (Gemmill et al, 2019). The leading cause of death among females 15–49 years old in the U.S. from 2015 to 2019 was unintentional injury; overdoses made up 61% of these injuries (CDC, 2022b). In Utah, the drug-induced pregnancy-associated mortality ratio in 2014 was 11.7 deaths per 100,000 live births (Smid et al, 2019). Few states have published SUD-related maternal mortality findings exploring risk factors and substance use patterns among SUD-related deaths.

In New Mexico (NM), the fatal overdose death rate increased from 24.0 deaths per 100,000 people in 2015 to 33.3 deaths per 100,000 people in 2019 (Bureau of Vital Records and Health Statistics, 2019). Additionally, NM had the highest age-adjusted alcohol-attributable death rate in the country from 2011 to 2015, with 53.1 deaths per 100,000 people compared to the national average of 28.0 deaths per 100,000 people (Esser et al, 2020). While NM rates of substance use and death due to substance use in the general population are high, experiences of pregnant and postpartum people who have Substance Use Disorder (SUD)-related deaths have not been previously evaluated in NM.

The aim of this study was to assess ratios of SUD maternal mortality and investigate risk factors associated with SUD-related and non-SUD-related deaths among all pregnancy-associated deaths in New Mexico from 2015 to 2019. This involved comparing demographics, pregnancy experiences, circumstances surrounding deaths, treatment of mental health conditions, and experiences of social stressors of SUD-related deaths to non-SUD-related deaths. We also explored patterns of substance use among both groups.

## Methods

### Population and Study Design

We conducted univariate analyses on a cohort of pregnancy-associated deaths in NM for the period 2015–2019 to assess the association between demographics, pregnancy factors, circumstances surrounding deaths, treatment of mental health conditions, and experiences with social stressors and experiencing a SUD-related death. We included in this analysis all pregnancy-associated deaths that occurred among NM residents between 2015 and 2019 that the New Mexico Maternal Mortality Review Committee (NM MMRC) reviewed. As defined by the CDC, pregnancy-associated deaths are deaths that occur during pregnancy or within one year of pregnancy from any cause; pregnancy-related deaths occur due to a pregnancy complication, a chain of events initiated by pregnancy, or the aggravation of an unrelated condition by the physiologic effects of pregnancy; and pregnancy-associated but not related deaths occur due to a cause that is not related to pregnancy (Smoots et al., 2021). This study received expedited approval by the New Mexico State University IRB.

We analyzed findings from case review and abstraction derived from birth and death certificates, medical and social service records, and law enforcement and autopsy reports to detail each person’s pregnancy, delivery, and circumstances surrounding the death. Each death was reviewed by NM MMRC members, including clinicians with a perinatal focus, substance use experts, public health and community health professionals, social workers, nurses, doulas, and community members with lived experiences related to maternal mortality. The NM MMRC reviewed the case narrative of each death and determined (1) primary causes of death; (2) contributing factors to and circumstances surrounding death; (3) preventability of death; and (4) pregnancy-relatedness of death.

### Outcome and Variables

Our objective was to describe prevalence of risk factors among people with pregnancy-associated deaths. Our outcome was a SUD-related death, defined as one where substance use was determined to be the primary cause of death, or where SUD was listed as a definite or probable contributing factor to the death by the NM MMRC. This ensured that all instances where substance use played a major role in the death were included, while excluding people with documented substance use that died in a manner entirely unrelated to the substance use. SUD-related deaths were not limited to overdoses and could include other scenarios such as methamphetamine-associated cardiomyopathy, suicide, and other mechanisms of injury. All other deaths were classified as non-SUD-related.

We included variables in our analysis that were abstracted for MMRC case review and, a priori, are relevant risk factors for maternal mortality – these are demographics, pregnancy factors, circumstances surrounding death, documented treatment of mental health conditions (MHC), indicators of social stressors, evidence of substance use, and types and number of substances used.

Certified Nurse Midwives and Nurses are the case abstractors in New Mexico. They are trained by the CDC on how to complete public health abstracting to ascertain data from birth certificates, death certificates, medical records, law enforcement reports, and autopsy reports. One abstractor reviews and codes each case and consults with the second abstractor as needed. All data are entered into the CDC Maternal Mortality Review Information Application (MMRIA) system. The CDC and New Mexico Data Analysts complete data checks for every case and provide abstractors with feedback on missing or inconsistent data entry. Data corrections are updated in MMRIA.

Demographics included year of death, age, race and ethnicity, education, marital status, geographic location of residence at time of death, and insurance status. Age categories included under 20 years old, 20–29 years old, 30–34 years old, and 35 years or older. Race and ethnicity categories included Hispanic, American Indian or Alaska Native, Black or African American, Non-Hispanic white, and Bi-racial. Education categories included less than high school education, high school graduate, some college, and college graduate. Marital status categories included married, divorced, or never married. Race and ethnicity, education, and marital status were sourced from birth certificates or fetal death certificates, or decedent’s death certificates if missing from infant certificates. We used the same categories that were used by these certificates. For race and ethnicity, we combined these two separate variables into one. Any person who was Hispanic was listed as Hispanic, and any person who was not Hispanic was listed as American Indian or Alaska Native, Black or African American, Non-Hispanic White, or Bi-Racial. Bi-Racial included any person who had multiple races selected. We classified the residence at time of death by categorizing the county in which the person lived into metropolitan (population of 50,000 or greater), micropolitan (10,000–49,999), and rural (fewer than 10,000) categories following the most recent National Center for Health Statistics Urban–Rural Classification Scheme (Ingram & Franco, 2014). Insurance statuses were sourced from birth or fetal death certificates, or prenatal care records if missing from certificates, and placed all people with Medicaid insurance into the Medicaid category, all people without Medicaid and with private insurance into Private, and people with other insurance types into Other, which exclusively included Medicare or self-pay.

Pregnancy factors included gravidity, number of prenatal care visits, trimester of first prenatal care visit, pregnancy outcome, and timing of death in relation to pregnancy. Pregnancy outcomes included died while pregnant; spontaneous abortion, therapeutic abortion, or ectopic pregnancy; and delivered. The latter included live births and stillbirths. Timing of death categories included Pregnant, 0–42 days after pregnancy, and 43–365 days after pregnancy. Circumstances surrounding the death, as determined by the MMRC, included pregnancy-relatedness, preventability of death, whether mental health was a contributing factor to death, whether death was a suicide, the cause of death, and the mechanisms of injury among injury and mental health deaths.

We assessed documentation of treatment for any MHC before, during, and after pregnancy, and during any of these time periods. MHCs with documentation of treatment was ascertained from any records available and included anxiety disorder, bipolar disorder, depression, SUD, psychotic disorder, or any MHC. Due to ambiguous documentation of these treatments, “No treatment” and missing responses were combined into one category for the MHC treatment analyses.

Social stressors were ascertained from any records available and included Child Protective Services (CPS) involvement; domestic violence; unemployment; previous suicide attempt; substance use treatment; recent trauma; childhood trauma; unwanted pregnancy; previous psychiatric hospitalization; and any social stressors, defined as having one or more of the above stressors.

### Substance Use Variables

Evidence of substance use in prenatal care documentation was determined by the abstractors based on their review of the prenatal care record. This was coded into a dichotomous variable. We additionally investigated types of substances used by each person at the time of death. We sourced this information from the toxicology report among cases with toxicology available. The New Mexico Office of Medical Examiners orders and completes all toxicology reports. We grouped substances into 11 categories: amphetamines, opiates/opioids, alcohol, benzodiazepines, marijuana, buprenorphine/naloxone/methadone, antidepressants/antipsychotics, anticonvulsants, tobacco, barbiturates, and other. Opiates/opioids included fentanyl, heroin, morphine sulfate, oxycodone hydrochloride, and oxymorphone hydrochloride. Other included Benadryl, Benztropine, caffeine, carbon monoxide, codeine free, meprobamate, detromethorphan, and sodium. Based on these data, we determined the extent of polysubstance use, defined as use of any two or more substances. We counted the number of substances documented per person to further explore the extent of polysubstance use among these cases. Due to polysubstance use the number of substances was more than the number of deaths, so we additionally calculated how many SUD-related and non-SUD-related deaths had at least one substance detected in their toxicology report, and how many people had a toxicology that was not positive for any substances.

### Statistical Analysis

We calculated the pregnancy-associated mortality ratio for all pregnancy-associated deaths that occurred in 2015–2019. We then calculated the SUD-related mortality ratio by using the number of SUD-related deaths for the numerator divided by the number of live births for the denominator for 2015–2019 deaths overall and per individual year using live birth counts from the New Mexico Indicator Based Information System (New Mexico Department of Health, 2022). We calculated 95% confidence intervals for these ratios and completed a linear regression to assess for differences in mortality ratios by year. We compared demographics, pregnancy factors, and circumstances surrounding the death among SUD-related and non-SUD-related deaths. We also compared treatment for different types of MHCs before, during, and after pregnancy among people with SUD-related and non-SUD-related deaths. We compared the experience of social stressors among people with SUD-related and non-SUD-related deaths. We performed univariate analyses of these risk factors using chi-square tests in Stata/MP 17.0 (Stata Corporation, College Station, TX) to assess the differences between SUD-related and non-SUD-related deaths. We used Fisher’s exact test for cases with cell sizes < 5. P-values of < 0.05 were considered statistically significant.

We examined evidence of substance use during prenatal care, types of substances used at the time of death, and polysubstance use.

## Results

**Fig. 1:**
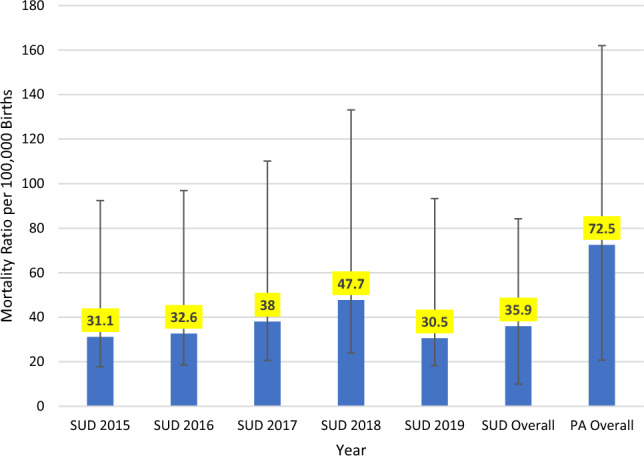
SUD-Related Mortality Ratio by Year and Overall, and Pregnancy-Associated (PA) Mortality Ratio Overall, per 100,000 Births, and Confidence Intervals in New Mexico by Year, 2015–2019

We assessed 87 pregnancy-associated deaths in New Mexico from 2015 to 2019. Of these deaths, 49% were SUD-related deaths (n = 43) and 51% were non-SUD-related deaths (n = 44). The pregnancy-associated mortality ratio for 2015–2019 was 72.5 deaths per 100,000 live births. The SUD-related mortality ratio was 35.8 deaths per 100,000 live births. The SUD-related mortality ratio varied from 2015 to 2018, with 31.1 (95% CI 13.4–61.3) deaths per 100,000 live births in 2015 to 47.7 (95% CI 23.8–85.4) deaths per 100,000 births in 2018 but decreased in 2019 to 30.5 (CI 12.3–62.8) deaths per 100,000 people. Our time trend findings were not statistically significant (p = 0.62) (Fig. 1).

People with SUD-related deaths were more likely to have died 43–365 days postpartum (81% vs. 45%, p-value = 0.002), have had mental health conditions as a contributing factor to their death (81% vs. 11%, p-value < 0.001), have had mental health conditions as their primary cause of death (47% vs 10%, p < 0.001), and have died of an overdose (41% vs. 8%, p-value = 0.002) compared to people with a non-SUD-related death (Table 1). We found no statistical differences in the other factors evaluated in Table 1. There were no statistically significant differences between SUD-related and non-SUD-related deaths in preventability (79% of all pregnancy-associated deaths in NM were preventable) or death from suicide. Among the SUD-related deaths, 54% of people were Hispanic, 95% were on Medicaid, 86% had delivered, and 17% were suicides.

**Table 1:** Demographic, pregnancy, and death characteristics of substance use disorder (SUD)-related deaths and non-SUD-related deaths in New Mexico, 2015–2019

	SUD case (n = 43) number^a^ (%)	Non-SUD case (n = 44) number^a^ (%)	P-value (bolded if < 0.05)
Year of death			0.53
2015	8 (19)	10 (23)	
2016	8 (19)	12 (27)	
2017	9 (21)	11 (25)	
2018	11 (26)	8 (18)	
2019	7 (16)	3 (7)	
Age			0.34
< 20	3 (7)	4 (9)	
20–29	17 (40)	25 (57)	
30–34	12 (28)	7 (16)	
35 +	11 (26)	8 (18)	
Race/ethnicity			0.10
Hispanic	23 (54)	14 (32)	
Non-Hispanic white	14 (33)	15 (34)	
American Indian or Alaska Native	6 (14)	12 (27)	
Black or African American	0	2 (5)	
Bi-racial	0	1 (2)	
Education			0.11
Less than high school	15 (35)	14 (32)	
High school graduate	16 (37)	11 (25)	
Some college	12 (28)	14 (32)	
College graduate	0	5 (11)	
Marital status			0.28
Married	8 (19)	13 (29)	
Divorced	7 (16)	3 (7)	
Never Married	28 (65)	28 (64)	
Residence at time of death			0.38
Metropolitan	29 (71)	25 (57)	
Micropolitan	11 (27)	16 (36)	
Rural	1 (2)	3 (7)	
Insurance			0.07
Medicaid	36 (95)	26 (76)	
Private	1 (3)	5 (15)	
Other	1 (3)	3 (9)	
Gravidity			0.55
1	7 (20)	6 (16)	
2	4 (11)	8 (21)	
3+	24 (69)	24 (63)	
Number of prenatal care visits			0.25
0–3	14 (36)	6 (17)	
4–8	9 (23)	11 (31)	
9–13	13 (33)	12 (34)	
14+	3 (8)	6 (17)	
Trimester of first prenatal care visit			0.27
1st	17 (40)	25 (58)	
2nd	8 (19)	8 (19)	
3rd	1 (2)	2 (5)	
None	3 (7)	0	
Pregnancy outcome			0.07
Died while pregnant	3 (7)	11 (25)	
Spontaneous abortion, therapeutic abortion, or ectopic pregnancy	3 (7)	3 (7)	
Delivered	37 (86)	30 (69)	
Timing of death			**0.002**
Pregnant	3 (7)	11 (25)	
0–42 days postpartum	5 (12)	13 (30)	
43–365 days postpartum	35 (81)	20 (45)	
Pregnancy relatedness			0.50
Pregnancy associated, not related	25 (58)	20 (45)	
Pregnancy related	12 (28)	16 (36)	
Unable to determine	6 (14)	8 (18)	
Preventable death			0.75
Yes	34 (79)	36 (82)	
No	9 (21)	8 (18)	
Mental health contributed to death			** < 0.001**
Yes/probably	35 (81)	5 (11)	
No	4 (9)	31 (70)	
Death was a suicide			0.37
Yes/probably	7 (17)	4 (9)	
No	29 (67)	36 (82)	
Cause of death			** < 0.001**
Mental health	20 (47)	4 (10)	
Injury	10 (23)	21 (50)	
Cardiac	5 (12)	2 (5)	
Other medical	4 (9)	1 (2)	
Infection	2 (5)	3 (7)	
Hemorrhage	1 (2)	3 (7)	
Embolism	1 (2)	5 (12)	
Mechanisms of injury			**0.002**
Overdose	12 (41)	2 (8)	
Motor Vehicle Crash	5 (17)	17 (65)	
Hanging	3 (10)	2 (8)	
Drowning	3 (10)	1 (4)	
Firearm	3 (10)	2 (8)	
Stabbing	1 (3)	0	
CO poisoning	1 (3)	0	
Assault	1 (3)	0	

^**a**^Columns may not add to total due to missing data

People with SUD-related deaths were more likely to have received treatment before pregnancy for anxiety disorder (37% vs. 11%, p-value = 0.005), depression (37% vs. 9%, p-value = 0.002), SUD (40% vs. 2%, p-value = 0.01), and any MHCs (61% vs. 16%, p-value < 0.001) compared to people with non-SUD-related deaths. People with SUD-related deaths were more likely to have received treatment for depression (23% vs. 7%, p-value = 0.04) after pregnancy compared to people with non-SUD-related deaths. They were also more likely to have received treatment for anxiety disorder (44% vs. 11%, p-value = 0.001), depression (47% vs. 11%, p-value < 0.001), SUD (49% vs. 2%, p-value < 0.001), or any MHCs (72% vs. 18%, p-value < 0.001) at any point before, during, or after pregnancy compared to non-SUD-related deaths. Of all people with SUD-related deaths, fewer than half received treatment for SUD before pregnancy (40%) or during pregnancy (37%). Fewer than one-third of people with a SUD-related death received SUD treatment after pregnancy (28%), while the majority of these SUD-related deaths occurred 43–365 days postpartum (Table 2).

**Table 2:** Comparison of documented treatment for mental health conditions before, during, or after pregnancy of SUD-related and non-SUD-related deaths in New Mexico, 2015–2019

	SUD cases (N = 43) number^a^ (%)	Non-SUD case (N = 44) number^a^ (%)	P-value (bolded if < 0.05)
Before pregnancy			
Anxiety disorder	16 (37)	5 (11)	**0.005**
Bipolar disorder	5 (12)	4 (9)	0.74
Depression	16 (37)	4 (9)	**0.002**
Substance use disorder	17 (40)	1 (2)	**0.01**
Psychotic disorder	0	0	–
Any mental health condition	26 (61)	7 (16)	** < 0.001**
During pregnancy			
Anxiety disorder	10 (23)	4 (9)	0.09
Bipolar disorder	2 (5)	2 (5)	1.00
Depression	9 (21)	4 (9)	0.14
Substance use disorder	16 (37)	0	–
Psychotic disorder	0	0	–
Any mental health condition	18 (42)	6 (14)	**0.004**
After pregnancy			
Anxiety disorder	9 (21)	3 (7)	0.07
Bipolar disorder	3 (8)	1 (2)	0.36
Depression	10 (23)	3 (7)	**0.04**
Substance use disorder	12 (28)	0	–
Psychotic disorder	1 (2)	0	–
Any mental health condition	19 (44)	5 (12)	**0.001**
Before, during, or after			
Anxiety disorder	19 (44)	5 (11)	**0.001**
Bipolar disorder	5 (11)	4 (9)	0.74
Depression	20 (47)	5 (11)	** < 0.001**
Substance use disorder	21 (49)	1 (2)	** < 0.001**
Psychotic disorder	1 (2)	0	–
Any mental health condition	31 (72)	8 (18)	** < 0.001**

^**a**^Columns may not add to total due to documented treatment for multiple mental health conditions

People with SUD-related deaths were more likely to have had CPS involvement (54% vs 14%, p-value = 0.01), have been unemployed (49% vs. 14%, p-value = 0.03) and were more likely to have experienced any social stressors (86% vs 30%, p < 0.001) compared to people with non-SUD-related deaths (Table 3). The most common stressors among people with SUD-related deaths were CPS involvement (54%), unemployment (49%), domestic violence (41%), and previous suicide attempt (32%). Fewer than one-third of people with SUD-related deaths had substance use treatment as a documented stressor (27%).

**Table 3:** Experiences of social stressors of SUD-related and non-SUD-related deaths among pregnancy-associated deaths in New Mexico, 2015–2019

	SUD case (n = 43) number (%)	Non-SUD case (n = 44) number (%)	P-value (bolded if < 0.05)
Children youth and family department involvement	20 (54)	2 (14)	**0.01**
Unemployment	18 (49)	2 (14)	**0.03**
Domestic violence	15 (41)	4 (29)	0.53
Previous suicide attempt	12 (32)	1 (7)	0.08
Substance use treatment	10 (27)	0	–
Previous psychiatric hospitalization	10 (27)	4 (29)	1.0
Childhood trauma	8 (22)	2 (14)	0.71
Unwanted pregnancy	6 (16)	1 (7)	0.66
Recent trauma	5 (14)	0	–-
Any social stressors	37 (86)	14 (30)	** < 0.001**

The substances most used at the time of death among SUD-related deaths were amphetamines (70%), opiates/opioids (35%), alcohol (30%), and benzodiazepines (26%). Seventy-four percent of all SUD-related deaths had at least one substance detected in their toxicology, compared to 30% of non-SUD-related deaths. Just under half (49%) of all SUD-related deaths had evidence of substance use documented during prenatal care. Most SUD-related deaths engaged in polysubstance use (63%), with 42% of SUD-related-deaths using three or more substances at the time of death (Table 4).

**Table 4:** Substance use among non-SUD-related and SUD-related deaths in New Mexico, 2015–2019

	SUD-related death (N = 43) number (%)	Non-SUD-related death (N = 44) number (%)
Substances detected in toxicology^a^		
Amphetamines	30 (70)	3 (7)
Opiates/opioids	15 (35)	5 (11)
Alcohol	13 (30)	4 (9)
Benzodiazepines	11 (26)	2 (5)
Marijuana	8 (19)	3 (7)
Buprenorphine/naloxone/methadone	7 (16)	1 (2)
Antidepressants/antipsychotics	4 (9)	3 (7)
Anticonvulsants	3 (7)	1 (2)
Tobacco	1 (2)	0
Barbiturates	1 (2)	1 (2)
Other	3 (7)	5 (11)
Toxicology not positive for substances	4 (9)	19 (43)
Toxicology not done	7 (16)	12 (27)
Evidence of substance use during prenatal care	21 (49)	9 (20)
No substances detected (0 substances)	11 (26)	31 (70)
At least one substance detected (1 + substances)	32 (74)	13 (30)
One substance detected	5 (12)	8 (18)
Two substances detected	9 (21)	2 (5)
Three or more substances detected	18 (42)	3 (7)
Polysubstance use (2 + substances)	27 (63)	5 (11)

^a^Numbers and percentages may not add to total due to polysubstance use

## Discussion

We found more SUD-related deaths among people with Medicaid insurance, and people with SUD-related deaths were more likely to die 43–365 days postpartum than people with non-SUD-related deaths. We additionally found a high frequency of treatment for MHCs, experiences of CPS involvement and unemployment as social stressors, polysubstance use, and a lack of SUD detected during prenatal care among people with SUD-related deaths.

Our finding that SUD-related deaths were more likely to occur 43–365 days postpartum is consistent with previous studies. An analysis of drug-induced deaths in Utah found that 80% of deaths occurred in this same “late postpartum” period of 43–365 days after pregnancy (Smid et al, 2019). Additionally, while high rates of prenatal abstinence are found across all substances, rates of return to use in the postpartum period are high—a study focused on opioid use found that rates of opioid relapse, overdose, and death increased in the year following a birth among people with Opioid Use Disorder (Schiff et al, 2018). An additional study of people with Opioid Use Disorder receiving buprenorphine treatment at 6 months postpartum found that 51.7% of people had substance use recurrence after delivery (Ellis et al, 2022). Qualitative studies have found that reasons for returning to use include stress of caring for a newborn, lack of sleep, lack of social and financial support, shame, low self-esteem, and experiences with domestic violence (Ripley-Moffitt et al, 2008; Notley et al, 2015; Pallatino et al., 2021). Return to substance use, particularly opioids including fentanyl, is dangerous as there is a higher risk of an overdose due to a reduced tolerance following abstinence (Warner-Smith et al, 2001). Previous studies have found that people with strong social supports, including having spouses or significant others that did not use drugs and having positive family relationships postpartum, were less likely to return to use after delivery (Ellis et al., 2004). Qualitative studies have also found that social support systems, such as online forums or peer support workers in perinatal substance use programs, can be helpful during treatment and in assisting patients to find continued social supports after leaving the programs (Moore et al, 2017; Olding et al, 2022). Expanding peer support networks may improve support and access to resources for people in their postpartum periods, which may reduce stress and likelihood of returning to use.

People represented in the SUD-related deaths were more likely to have been covered by Medicaid for healthcare in the perinatal period. In many states—including NM, until 2022—Medicaid for pregnant people only covered 60 days postpartum, and up to one-third of postpartum people have faced disruptions in insurance statuses (Shah & Friedman, 2022). Uninsured people in the postpartum period face poorer outcomes, particularly in treating chronic diseases—a known contributor to maternal mortality (Villavicencio et al, 2020).

New Mexico pregnancy-related Medicaid was extended in April 2022 to cover people up to one year postpartum. Implementation of Medicaid extension beyond 60 days in Texas resulted in an increase in uptake of mental and behavioral health services (Wang et al, 2022). Previous studies have found that, as the Medicaid eligibility threshold increased to include more people, SUD-related deaths reduced by 6.5% from 2002 to 2015, and states that expanded Medicaid mental health plans found increases in enrollment in treatment programs (Snider et al, 2019). Based on results of expansion of Medicaid, we expect to see similar improvements in maternal outcomes due to Medicaid extension. Until we complete case reviews for 2022 deaths, we are not able to determine the impact of Medicaid extension on preventing deaths; however, we hope to see an increase in receipt of SUD treatment and a reduction in preventable, SUD-related deaths for our at-risk population of people who are on Medicaid and between 43 and 365 days postpartum.

We found a greater likelihood of treatment for MHCs and experiences of social stressors among people with SUD-related deaths compared to people with non-SUD-related deaths. Previous studies have found high rates of depression, anxiety, and other MHCs among people with SUD-related deaths and pregnant people with SUD (Arnaudo et al, 2017; Smid et al, 2019). CPS involvement and unemployment were leading social stressors, followed by domestic violence and a previous suicide attempt. Experiences of chronic stress can increase rates of substance use, particularly among people with comorbidities of MHCs and SUD, and among people with experiences of abuse or violence (Brady et al., 2005; Sinha, 2008). Substance use treatments must be trauma-informed and take into consideration the common co-occurring morbidities to address all aspects of the person’s health comprehensively and effectively, rather than exclusively focusing on clinical or acute addiction issues.

We found that fewer than half of people (49%) with SUD-related deaths received SUD treatment at any point before, during, or after pregnancy. Universal screening of all people at their first prenatal visit with a validated tool, following The American College of Obstetricians and Gynecologists (ACOG) guidelines, could reduce stigma often associated with substance use during pregnancy. It would also facilitate receipt of opioid agonist pharmacotherapy—the therapy recommended by ACOG, a component of Medication Assisted Treatment (MAT) and Medications for Opioid Use Disorder (MOUD)—and support to pregnant people using substances prenatally and in the postpartum period (ACOG Committee Opinion, 2017). According to a New Mexico Department of Health Gap Analysis in 2020 on Substance Use Treatment, only 38% of SUD treatment locations in New Mexico offered MAT, and eight counties out of 33 did not have any facility that offered this treatment. Additionally, many facilities reported that they rely on one provider to prescribe MAT and stated that patients will often cancel appointments because they cannot afford co-pays. Of 308 facilities in New Mexico, 240 (78%) accepted pregnant patients, and 51 (17%) treatment locations allowed parents to bring a child with them to sessions or transitional living houses (New Mexico Department of Health, 2020). This indicates that there are treatment resources for pregnant people, but not as many that provide MAT, the more effective treatment, and fewer that accept parenting individuals. New Mexico needs to increase the number of providers that have the certification required to prescribe MAT so that it is more accessible, particularly for pregnant and postpartum people and people in rural areas with fewer treatment options. New Mexico also needs to expand affordable treatment options with reduced co-pays to improve patient retention.

A majority of people with SUD-related deaths engaged in polysubstance use (63%). These findings are consistent with previous studies that have found between 64 and 89% of pregnant people with Opioid Use Disorder engaging in polysubstance use (Jarlenski et al, 2017, 2020; Smid et al, 2019). Treatment programs must account for polysubstance use to treat SUD more effectively.

While amphetamines and opiates/opioids were the most used substances, alcohol made up 30% of substances used among SUD-related deaths. This is consistent with findings that NM has had the highest age-adjusted alcohol attributable death rate in the country. As treatment for other SUDs are highlighted amidst the opioid crisis, treatment for alcohol use disorder still must remain a priority for SUD treatment in NM.

Just under half of SUD-related deaths (49%) had any evidence of substance use in their prenatal care record, indicating that patients were either not using substances during pregnancy but still had a SUD-related death, providers were unaware of their patient’s substance use during pregnancy, or that these patients did not receive any prenatal care. Providers may be unaware of substance use if a patient does not disclose information on their use, possibly due to a fear of stigma or punishments such as CPS involvement, child separation, or legal ramifications (Falleta et al., 2018; Stone, 2015).

Data-Driven Recommendations to Prevent SUD-Related Deaths

- Expand peer support networks to improve access to resources in the postpartum period.
- Address mental health conditions and other common co-occurring morbidities by implementing trauma-informed substance use treatments.
- Account for polysubstance use in treatment programs.
- Implement universal screening of all people at their first prenatal visit with a validated tool to reduce stigma often associated with substance use during pregnancy.
- Increase the number of providers able to prescribe MAT, particularly for pregnant and postpartum people and people in rural areas in NM.
- Expand affordable treatment options with reduced co-pays in NM to improve patient retention.

## Limitations

Our study had several limitations. We had a small sample size of 87 deaths total, with 43 SUD-related deaths, and this may have limited our ability to detect statistically significant differences and draw definitive conclusions from our findings. Our small sample size restricted us to descriptive, univariate analyses. These findings are not generalizable to all birthing people in New Mexico, or to all people who die SUD-related deaths. These data represent deaths among pregnant and postpartum people with SUD in New Mexico and may not be generalizable to other state or national pregnant and postpartum populations.

Inconsistency of data entry and overall missingness of data may have contributed to misclassification in our analyses. If there was no documented treatment for a MHC, abstractors left the variable blank, meaning that the missing category includes both people with unknown documentation of MHCs and people who were known to be not treated for MHCs.

Nearly one-third (27%) of non-SUD-related deaths did not have toxicology completed, either because an autopsy was not completed, or the Medical Investigator did not deem one necessary. This amount of missing data limits what conclusions we can draw from these findings.

Additionally, information on treatments received for SUD—such as MAT, counseling, and inpatient or outpatient treatment—the duration of treatment, dosage of medications prescribed, or other information indicating resources pregnant and postpartum people accessed was ultimately unavailable and thus limited our conclusions on these topics.

## Conclusions for Practice

Substance use is a leading contributing factor to death in the United States. SUD-related deaths are more likely to occur postpartum, and nearly half of those who died a SUD-related death in NM were not properly screened or detected for SUD when receiving prenatal care. Providers, health departments, and community organizations must prioritize support and treatment for people using substances both during and after pregnancy to prevent death and improve quality of life for pregnant and postpartum people and their families.

## Data Availability

Data reside with study authors, New Mexico Maternal Mortality Review Committee abstractors and analysts, and the New Mexico Department of Health. Researchers may apply to view the data.

## Code Availability

STATA code is available upon request.
